# Wild-type huntingtin ameliorates striatal neuronal atrophy but does not prevent other abnormalities in the YAC128 mouse model of Huntington disease

**DOI:** 10.1186/1471-2202-7-80

**Published:** 2006-12-05

**Authors:** Jeremy M Van Raamsdonk, Jacqueline Pearson, Zoe Murphy, Michael R Hayden, Blair R Leavitt

**Affiliations:** 1 Department of Medical Genetics and Centre for Molecular Medicine and Therapeutics, Child and Family Research Institute, University of British Columbia, Vancouver, V5Z 4H4, BC, Canada

## Abstract

**Background:**

Huntington disease (HD) is an adult onset neurodegenerative disorder caused by a polyglutamine expansion in the huntingtin (htt) protein. Htt function is essential for embryonic survival as well as normal function during the postnatal period. In addition to having roles in transcription and transport, recent evidence demonstrates that wild-type htt is neuroprotective *in vivo*. To determine whether treatment with wild-type htt would be beneficial in HD, we crossed the YAC128 mouse model of HD with mice that over-express wild-type htt (YAC18 mice) to generate YAC128 mice that over-express wild-type htt (YAC18/128 mice).

**Results:**

YAC18/128 mice were found to express mutant htt at the same level as YAC128 mice and wild-type htt at the same level as YAC18 mice. YAC18/128 mice show no significant behavioural improvement compared to YAC128 mice in the rotarod test of motor coordination or in an automated open field test. In the brain, YAC18/128 mice show no significant improvement in striatal volume, striatal neuronal numbers or striatal DARPP-32 expression compared to YAC128 mice. In contrast, striatal neuronal cross-sectional area showed significant improvement in YAC18/128 mice compared to YAC128 mice.

**Conclusion:**

While the over-expression of wild-type htt results in a mild improvement in striatal neuropathology in YAC128 mice, our findings suggest that treatment with wild-type htt may not be sufficient to ameliorate the symptoms of HD in this model.

## Background

Huntington disease (HD) is an autosomal dominant disorder resulting from a trinucleotide CAG expansion in the HD gene. While the expression of mutant htt is sufficient to cause HD-like symptoms with normal expression levels of wild-type htt [1-3], recent data suggests that decreased levels of wild-type htt in HD patients may also contribute significantly to the pathogenesis of HD [4]. In support of this, we have recently demonstrated that the loss of wild-type htt in YAC128 mice significantly worsens motor performance, survival and striatal neuronal size [5].

Htt function is essential for development as mice homozygous for the targeted inactivation of the mouse HD gene are embryonic lethal [6-8]. Furthermore, decreasing htt levels by 50% or more from birth results in neurological abnormalities [7,9,10]. The expression of wild-type htt is also essential postnatally as mice expressing decreased levels of wild-type htt in the forebrain beginning at postnatal day 5 were shown to have a progressive neurological phenotype [11]. Clearly, decreased wild-type htt levels alone can lead to phenotypic abnormalities independent of mutant htt.

As the functions of wild-type htt become more clear, it seems that one of the most critical functions for htt may be in promoting neuronal survival. *In vitro *studies have demonstrated that the over-expression of wild-type htt protects cells against various insults including 3-nitroproprionic acid, a toxin which damages the striatum and has been used to model HD [12]. It has also been shown that over-expression of wild-type htt can specifically protect against polyglutamine toxicity *in vitro *[13]. This finding has been extended *in vivo *where increased expression of wild-type htt eliminated apoptotic degeneration in the testis caused by the expression of mutant htt [5,14]. The mechanism by which htt protects neurons may be direct by sequestration of the pro-apoptotic protein HIP-1 [15] or indirectly mediated through htt's effect on expression and transport within the cell as htt has been shown to be involved in both the transcription and movement of the brain derived neurotrophic factor [16-18]. In contrast, fragments of mutant htt have been shown to also protect against some forms of injury (excitotoxicity) through a different mechanism, likely the induction of a stress response [19].

In HD, there is evidence that striatal neurons die through excitotoxic mechanisms [20,21]. As such, we have previously examined the ability of htt to protect neurons *in vivo *against two different excitotoxic neurotoxins. For these experiments, we used YAC18 mice that over-express htt from a yeast artificial chromosome containing the entire human HD gene with 18 CAG repeats [22]. After intraperitoneal injection of kainic acid, YAC18 mice showed dramatically less neuronal loss in the hippocampus compared to WT controls [42]. Similarly, YAC18 mice showed significant, htt dose-dependent protection against lesions caused by intrastriatal injection of quinolinic acid [23].

Based on the clear demonstration of *in vivo *protection against excitotoxic cell death in YAC18 mice and the general protective effect of wild-type htt that has been demonstrated *in vitro*, we designed this experiment to determine if the over-expression of wild-type htt would be beneficial in treating HD. Since HD patients have at least a 50% genetic reduction in wild-type htt levels (resulting from the presence of at least one copy of the mutant HD gene), it is plausible that this decrease in htt's neuroprotective function will make neurons more susceptible to the toxicity of mutant htt. A decrease in wild-type htt levels may also affect htt's role in transcription and transport within the cell [16-18,24]. In fact many of htt's assayable functions have been shown to be disrupted by polyglutamine expansion [12,16,17,23] and polyglutamine expansion also alters htt's ability to interact with its interacting proteins [24]. Thus, treatment of HD with wild-type htt may be beneficial by compensating for the loss of wild-type htt function and or through htt's general neuroprotective effect.

For this experiment we used the YAC128 mouse model of HD which recapitulates many aspects of the human disease [3,25]. These mice exhibit progressive motor dysfunction, cognitive impairment and selective neurodegeneration. YAC128 mice were crossed to YAC18 mice (which over-express full-length wild-type htt) to generate YAC128 mice that over-express wild-type htt (YAC18/128 mice). We show that over-expression of wild-type htt in YAC128 mice results in a mild improvement in striatal neuropathology but does not improve motor dysfunction.

## Results

### Generation of YAC128 mice that over-express wild-type huntingtin

**Figure 1:**
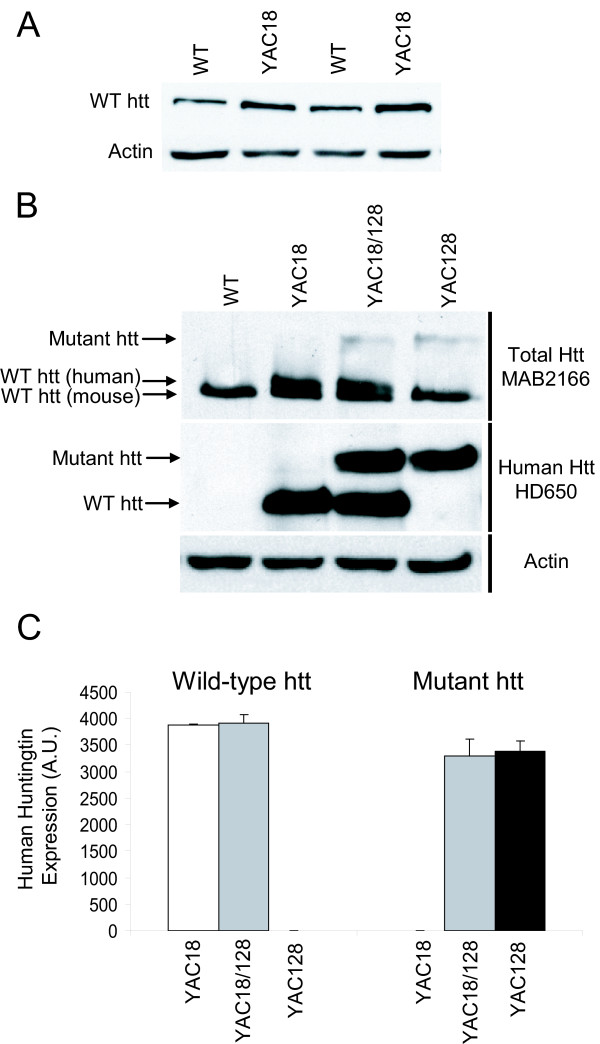
**Huntingtin expression in YAC18/128 mice**. YAC18 and YAC128 mice were crossed to generate YAC18/128 mice. **A. **To confirm high levels of htt over-expression in YAC18, line 212 mice we performed Western blots on whole brain lysates. We found that line 212 mice have 2.4 times the levels of wild-type htt as WT mice (WT: 0.386 ± 0.014 arbitrary units, YAC18: 0.942 ± 0.056 arbitrary units, p = 0.02). **B. **Total htt and human htt levels were assessed by Western blotting with MAB2166 and HD650 antibody respectively. The human specific HD650 antibody was used to detect htt expressed from the YAC transgenes in YAC18, YAC128 and YAC18/128 mice. Western blots performed with HD650 antibody indicate that YAC18/128 mice express both wild-type htt and mutant htt from YAC transgenes. As expected WT mice express no human htt, YAC18 mice express only wild-type human htt and YAC128 mice express only mutant human htt. Examination of total htt levels with MAB2166 antibody reveals that all mice express similar levels endogenous wild-type htt. **C. **Quantification of protein expression reveals that YAC18/128 mice express mutant htt at the same level as YAC128 mice (YAC128: 3389 ± 197 arbitrary units, YAC18/128: 3303 ± 316 arbitrary units, p = 0.8). N = 3 mice per group. Error bars indicate standard error of the mean. A.U. = arbitrary units.

To determine whether wild-type htt could protect against mutant htt toxicity in the brain, we crossed YAC128 mice with YAC18 mice to generate YAC128 mice that over-express wild-type htt (YAC18/128 mice). In order to maximize the amount of neuroprotection imparted by over-expression of wild-type htt, we used the highest expressing YAC18 line (line 212) available. We have previously shown that YAC18 line 212 mice express wild-type htt at 2–3 times endogenous levels [22,23,26] and exhibit the greatest degree of neuroprotection against quinolinic acid toxicity of all the YAC18 lines [23]. To confirm the high expression of htt in line 212 mice, we examined total wild-type huntingtin in line 212 and WT mice by Western blotting with polyclonal bkp1 antibody [27]. As previously reported, we found that line 212 mice express wild-type htt at levels that are more than two times the level of wild-type htt expression in WT mice (Fig. 1A, (WT: 0.386 ± 0.014 arbitrary units, YAC18: 0.942 ± 0.056 arbitrary units, p = 0.02).

After breeding YAC18 and YAC128 mice together, YAC18/128 mice were generated in equal proportions to WT, YAC18 and YAC128 mice indicating normal embryonic survival. Since the YAC transgenes used to generate YAC18 and YAC128 mice express human htt, we examined transgenic htt expression with the human specific htt antibody, HD650 (Fig. 1B; [3]). We also examined total htt expression using MAB2166 which detects both mouse and human htt (Fig. 1B). As expected, WT mice (non-transgenic FVB/N) express wild-type mouse htt and no human htt. YAC18 mice express increased levels of wild-type htt which are accounted for by increased human htt expression. YAC128 mice express wild-type htt at the same level as WT mice and human mutant htt. Finally, YAC18/128 mice express increased levels of wild-type htt and mutant human htt. Importantly, the level of mutant htt expression was equal between the YAC128 and YAC18/128 mice indicating that the over-expression of wild-type htt did not down-regulate the expression of mutant htt (Fig. 1C; YAC128: 3389 ± 197 arbitrary units, YAC18/128: 3303 ± 316 arbitrary units, p = 0.8; N = 3).

### Over-expression of wild-type huntingtin does not improve motor function in YAC128 mice

To examine the effect of wild-type htt on the motor dysfunction present in the YAC128 mice, we monitored motor coordination on the rotarod from 2 to 12 months of age. While ANOVA revealed an overall effect of genotype on rotarod performance (genotype: F_(3,36) _= 7.9, p < 0.001, N = 8 WT, 9 YAC128, 16 YAC18, 8 YAC18/128), there were no significant differences between the YAC128 mice and YAC18/128 at any time point (YAC128: 147 ± 13 seconds, YAC18/128: 134 ± 14 seconds, p^YAC128vsYAC18/128 ^= 0.5, YAC18: 164 ± 19 seconds). Both groups performed significantly worse than WT mice (WT: 227 ± 15 seconds, p < 0.001).

We have previously reported early hyperactivity and late hypoactivity in YAC128 mice compared to WT mice [3,5]. As such, we compared the activity of YAC18/128 mice and YAC128 mice at 2 and 12 months of age to determine if wild-type htt expression could ameliorate the abnormal activity pattern present in YAC128 mice. We observed no significant improvement in activity in YAC18/128 mice compared to YAC128 mice (2 months – YAC128: 334 ± 13 beam breaks, YAC18/128: 320 ± 12 beam breaks, p^YAC128vsYAC18/128 ^= 0.5, YAC18: 324 ± 11 beam breaks; 12 months – YAC128: 274 ± 19 beam breaks, YAC18/128: 289 ± 15 beam breaks, p^YAC128vsYAC18/128 ^= 0.5, YAC18: 277 ± 11; N = 8 WT, 9 YAC128, 16 YAC18, 8 YAC18/128). Overall, increasing wild-type htt expression did not provide a significant behavioural benefit to YAC128 mice.

While this study was not powered to demonstrate significant differences in survival, the number of YAC18/128 mice surviving to 12 months was similar to what we normally observe in YAC128 mice [Table 1; 12 month survival – WT males: 91%, YAC128 males: 73%, YAC18/128 males: 73% (8 of 11 mice), WT females: 90%, YAC128 females: 93%, YAC18/128 females: 100% (4 of 4 mice)].

Mice surviving to 12 months of age were sacrificed and we examined brain and testicular weight as these have been shown to be decreased in YAC128 mice [3,5]. In both cases, there was a significant difference between YAC128 and WT mice which was not improved by the over-expression of wild-type htt (Brain weight – WT: 403 ± 7 mg, YAC18: 402 ± 4 mg, YAC128: 384 ± 4 mg, YAC18/128: 382 ± 3 mg, p^YAC128vsYAC18/128 ^= 0.6, N = 17 WT, 17 YAC128, 14 YAC18, 17 YAC18/128; Testis weight – WT: 164 ± 5 mg, YAC18: 152 ± 7 mg, YAC128: 142 ± 5 mg, YAC18/128: 123 ± 4 mg, p^YAC128vsYAC18/128 ^= 0.01, N = 7 WT, 8 YAC128, 10 YAC18, 10 YAC18/128). Unexpectedly, the testicular weight in YAC18/128 mice was significantly less than in YAC128 mice and there was a trend towards decreased testicular weight in YAC18 mice compared to WT. This suggests the possibility that high levels of htt expression may be detrimental in the testis.

### Over-expression of wild-type huntingtin results in mild improvement in striatal neuropathology in YAC128 mice

**Figure 2:**
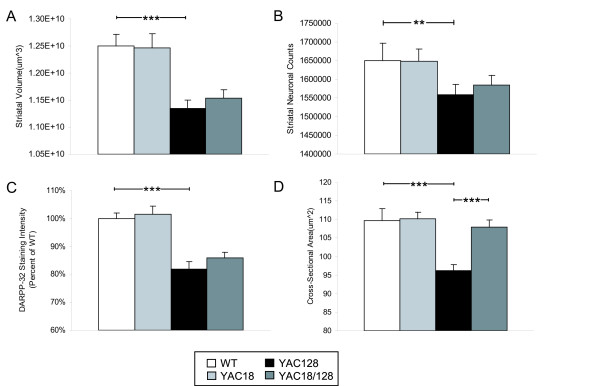
**Over-expression of wild-type htt results in mild improvements in striatal neuropathology in YAC128 mice**. Comparison of striatal phenotypes between YAC128 and YAC18/128 mice revealed that over-expression of wild-type htt resulted in no significant change in striatal volume (**panel A**: YAC128: 11.3 ± 0.2 mm^2^, YAC18/128: 11.6 ± 0.2 mm^2^, p = 0.3), striatal neuronal counts (**panel B**: YAC128: 1.56 ± 0.03 million neurons, YAC18/128: 1.59 ± 0.03 million neurons, p = 0.4) or striatal DARPP-32 expression (**panel C**: YAC128: 828 ± 28 arbitrary units, YAC18/128: 868 ± 22 arbitrary units, p = 0.3). In contrast, over-expression of wild-type htt resulted in a significant improvement in striatal neuronal cross-sectional area (**panel D**: YAC128: 96.2 ± 1.6 um^2^, YAC18/128: 108 ± 1.9 um^2^, p < 0.001). For each outcome measure, YAC128 mice show a significant deficit compared to WT mice. N = 17 WT, 17 YAC128, 14 YAC18, 17 YAC18/128 except for striatal DARPP-32 expression where N = 10 WT, 12 YAC128, 10 YAC18, 8 YAC18/128. Error bars show standard error of the mean. ** p < 0.01. *** p < 0.001.

YAC128 mice demonstrate clear striatal neuropathology at 12 months of age with decreased striatal volume, striatal neuronal loss, striatal neuronal atrophy and decreased striatal DARPP-32 expression [3,28]. To determine whether the expression of wild-type htt could ameliorate these striatal phenotypes we examined the striata of YAC18/128 mice. Comparing YAC18/128 mice and YAC128 mice revealed no significant improvement in striatal volume (Fig. 2A; YAC128: 11.3 ± 0.2 mm^3^, YAC18/128: 11.6 ± 0.2 mm^3^, p^YAC128vsYAC18/128 ^= 0.3, YAC18 = 12.5 ± 0.3 mm^3^; N = 17 WT, 17 YAC128, 14 YAC18, 17 YAC18/128), striatal neuronal numbers (Fig. 2B; YAC128: 1.56 ± 0.03 million neurons, YAC18/128: 1.59 ± 0.03 million neurons, p = 0.4, YAC18: 1.64 ± 0.03 million neurons; N = 17 WT, 17 YAC128, 14 YAC18, 17 YAC18/128) or striatal DARPP-32 expression (Fig. 2C; YAC128: 828 ± 28 arbitrary units, YAC18/128: 868 ± 22 arbitrary units, p^YAC128vsYAC18/128 ^= 0.3, YAC18: 1012 ± 19 arbitrary units; N = 10 WT, 12 YAC128, 10 YAC18, 8 YAC18/128) with increased wild-type htt expression. In each case, YAC128 mice showed significant abnormalities compared to WT mice. In contrast, increasing wild-type htt expression in YAC128 mice resulted in a significant reduction in striatal neuronal atrophy (Fig. 2D; YAC128: 96.2 ± 1.6 um^2^, YAC18/128: 108 ± 1.9 um^2^, p^YAC128vsYAC18/128 ^< 0.001, YAC18: 110 ± 1.7 um^2^; N = 17 WT, 17 YAC128, 14 YAC18, 17 YAC18/128) with the striatal neuronal cross-sectional area in YAC18/128 mice being almost restored to wild-type (WT: 110 ± 3 um^2^, p = 0.6).

## Discussion

Based on recent work demonstrating a neuroprotective function of wild-type htt and suggestions that loss of wild-type htt function contributes to HD pathogenesis, we investigated the therapeutic potential of wild-type htt in the YAC128 mouse model of HD. We found that over-expression of wild-type htt in YAC128 mice resulted in a mild improvement in striatal neuropathology with no significant improvement in behavioural phenotypes.

The effect of over-expression of wild-type htt in YAC128 mice is summarized in table 2. A robust finding of this study was that over-expression of wild-type htt in YAC128 mice restored striatal neuronal size. Similarly, we have found that decreasing wild-type htt levels in YAC128 mice results in decreased neuronal size [5]. Combined, these results suggest that wild-type htt levels influence neuronal size and suggest that loss of wild-type htt may contribute to the striatal neuronal atrophy observed in HD. An alternate possibility is that striatal neuronal size is more responsive to mildly beneficial effects of treatments as this measure has been shown to exhibit the most dramatic improvements in therapeutic trials in mouse models of HD [29-31]. The effect of wild-type htt on neuronal size may be related to htt's ability to increase BDNF transcription [17] and transport [16] since BDNF promotes the survival and differentiation of striatal neurons.

In contrast to the significant improvement in striatal neuronal size, there was no significant effect of wild-type htt over-expression on open field activity, striatal volume, striatal neuronal counts or striatal DARPP-32 levels in YAC128 mice, despite a trend towards improvement. In parallel with these experiments we examined the effect of eliminating wild-type htt expression in YAC128 mice and found that there was a trend towards decreased striatal volume, striatal neuronal counts and striatal DARPP-32 expression which did not reach significance [5] (see Additional file 1 for summary). It is possible that the 12 month time point chosen to assess neuropathology in these experiments was too late in the disease process and that differences in severity were masked by a ceiling effect or that we have missed differences in the onset of striatal neuropathology.

In our previous study we observed a significant increase of both rotarod performance and survival when wild-type htt levels were increased in YAC128 -/- mice to wild-type levels [5]. In this experiment, we did not observe any further improvement in either rotarod performance or survival with the over-expression of wild-type htt suggesting that there may be ceiling effect for the amount that wild-type htt can improve these outcome measures. While this study did not have enough power to demonstrate a significant improvement in survival, the fact that the percentage of mice surviving to 12 months was similar to YAC128 mice suggests that the over-expression of wild-type huntingtin does not have a dramatic effect on survival in YAC128 mice. The survival deficit in the YAC128 and YAC18/128 mice were only observed in male mice thus confirming our previous observations [5]. Unexpectedly, we found that increasing wild-type huntingtin expression in YAC128 mice resulted in further decreases in testicular mass. Combined with a trend towards decreased testicular mass in YAC18 mice, this suggests the possibility that expression of htt beyond a certain threshold may result in testicular atrophy which is exacerbated by the presence of mutant htt. It is also possible that the human origin of the over-expressed htt contributes to the testicular phenotype. Unfortunately, we are not aware of a mouse model that over-expresses wild-type mouse htt at high levels that would permit testing of this hypothesis.

In this study, we used the YAC128 mouse model of HD which transgenically expresses mutant huntingtin at approximately 75% of endogenous levels [3]. These mice have two intact copies of the wild-type HD gene and we have previously shown that they express wild-type htt at the same level as WT mice [5]. As such, YAC128 mice express higher levels of wild-type htt protein than patients with HD. The fact that the levels of wild-type htt are already increased in YAC128 mice may diminish the therapeutic benefit we observe in YAC18/128 mice by further over-expressing wild-type htt. To more directly assess the therapeutic benefit of wild-type htt in HD, one could over-express mouse wild-type htt in a knockin mouse model of HD to assess the effect of wild-type htt on early disease phenotypes in these mice.

The results of this study are congruent with comparisons of homozygous and heterozygous HD patients and HD mouse models which suggest that mutant htt has a greater influence on the disease phenotype than wild-type htt. Examination of disease severity in patients homozygous and heterozygous for mutations in the HD gene have reported either no difference or that homozygous HD patients are more severely affected [32-37]. Two independent studies have also examined the phenotype of mice that are homozygous for a targeted expansion of the HD gene (HD knock-in mice). In both cases, homozygous HD knock-in mice exhibited a more severe phenotype than heterozygous HD knock-in mice, but the differences were mild [38,39]. These studies suggest that mutant htt maintains many of the critical functions of wild-type htt as replacement of wild-type htt with mutant htt has only a mild effect on phenotype. However, it has been shown that polyglutamine expansion disrupts htt's neuroprotective function [12,23], at least part of htt's role in transcription [17] and transport [16], and also affects the interaction of htt with its interaction partners [24]. Since increasing mutant htt expression alone is known to result in a more severe phenotype in mice [40], it suggests that the increase in phenotypic severity between heterozygous and homozygous patients and animal models is mainly caused by the increase in mutant htt levels which is in line with our findings that increasing levels of wild-type htt has only a small impact on the disease phenotype.

These findings are surprising given the importance of htt function and its demonstrated neuroprotective abilities. Htt is essential for embryonic development and decreases in htt expression alone lead to abnormal phenotypes [6-8,10,11,41]. Further, htt has been shown to protect cells from death both *in vitro *and in the testis and in the brain [12-14,23]. While htt has been shown to specifically protect against polyglutamine toxicity both *in vitro *[13] and in the testis [5,14], our findings here indicate a milder protective effect against mutant htt toxicity in the brain. Given that wild-type htt exhibits protection against excitotoxic neurotoxins [23], our finding that wild-type htt mildly improves striatal neuropathology in YAC128 mice is not inconsistent with excitotoxicity contributing to the pathogenesis of HD.

## Conclusion

Overall, our results demonstrate that the over-expression of wild-type htt in YAC128 mice results in a mild improvement in striatal neuropathology. Based on the clear effect of htt over-expression on striatal neuronal size, it appears that htt function may be important in maintaining neuronal health. Despite the protective function of wild-type htt, our results suggest that mutant htt toxicity is primarily responsible for the pathognomic striatal neuropathology in HD and that treatment of HD with wild-type htt may not be sufficient to ameliorate the symptoms of the disease.

## Methods

### Mice

YAC18 and YAC128 mice that express human wild-type or mutant htt from a yeast artificial chromosome and wild-type littermates were used for these experiments [3,22]. For YAC18 mice, we used the high-expressing line 212 which expresses wild-type htt at 2–3 times endogenous levels [22]. Mice were maintained on the FVB/N (Charles River, Wilmington, MA) background strain. Mice were group housed with a normal light-dark cycle (lights on at 6:00 AM, lights off at 8:00 PM) in a clean facility and given free access to food and water. All experiments were carried out in accordance with protocols approved by the UBC Committee on Animal Care and the Canadian Council on Animal Care. The results shown are the combined results from male and female mice. We examined the data for both sexes separately and found no differences from the combined results.

### Behavioural analysis

Motor coordination was assessed on an accelerating rotarod (UGO Basile, Comerio, Italy) as previously described [5]. After training at 2 months, mice were tested bimonthly from 2 to 12 months of age with a maximum score for each trial of 300 seconds. Open field activity was assessed in an automated open field apparatus (San Diego Instruments, San Diego, California). Activity was measured at 2 months and at 12 months during a ten minute open field trial. Activity was measured as the number of beam crosses in the trial. Clean cages were used for each trial. For behavioural assessment we used 8 WT (5 F, 3 M), 9 YAC128 (5 F, 4 M), 16 YAC18 (9 F, 7 M) and 8 YAC18/128 (3 F, 5 M) mice.

### Western blotting

Protein levels were measured from homogenized whole brain lysates from a total of 3 mice per genotype. A low-bis acrylamide gel was run with 100 μg of total protein per sample for a total of 600 volt-hours. Proteins were transferred to a membrane at 24 volts for 1.5 hours. Blots were then probed with antibodies for either total htt (MAB2166, Chemicon, Temecula, California) or specifically human htt [HD650, (Slow et al., 2003a)] followed by an anti-mouse, peroxidase-conjugated secondary antibody before enhanced chemiluminescent detection. Protein levels were quantified using Quantity One software (Biorad, Hercules, CA).

### Neuropathology

Neuropathology was carried out on 17 WT (11 F, 6 M), 17 YAC128 (10 F, 7 M), 14 YAC18 (8 F, 6 M) and 17 YAC18/128 (8 F, 9 M) mice. Mice were perfused with 3% paraformaldehyde in phosphate buffered saline. Brains and testis were post-fixed in 3% paraformaldehyde for 24 hours and then equilibrated with PBS prior to weighing. Subsequently, brains were infiltrated with sucrose (25% in PBS), frozen and sectioned on a cryostat (Microm HM 500 M, Richard-Allan Scientific, Calamazoo, Michigan).

A series of 25 μm coronal sections spaced 200 μm apart were stained with NeuN primary antibody (1:100 dilution in 5% NGS, 0.1% T-X-100, PBS; Chemicon) overnight at room temperature, biotinylated anti-mouse secondary antibody (1:200 dilution in 1% NGS, 0.1% T-X-100, PBS) for 2 hours at room temperature and incubated in ABC reagent (ABC Elite kit, Vector) for 2 hours at room temperature before detection with metal-enhanced DAB solution (Pierce, Rockford, Illinois).

Striatal volume was determined using Stereoinvestigator software (Microbrightfield, Williston, Virginia). Briefly, the perimeter of the striatum was traced using a 2.5X objective in each section of the coronal series and the software calculated the volume of the entire structure. Subsequently, neuronal profiles in a 25 μm × 25 μm counting frame were counted with a 550 μm by 550 μm grid for all grids the fell within the outlined areas. The counts were then extrapolated to estimate the total number of neurons in the striatum. To determine neuronal cross-sectional areas, a single matched section from each animal was stained with an Alexa488-conjugated NeuN antibody (Chemicon). Mounted sections were analyzed using Stereoinvestigator to outline the perimeter of all clearly defined neurons within a 550 μm × 550 μm grid of 25 μm × 25 μm counting frames with the 100X objective. On average 32 neurons per mouse were assessed for a total of more than 450 neurons per genotype.

For measurement of DARPP-32 expression, sections were stained with rabbit anti-DARPP-32 antibody (Chemicon AB 1656, 1:1000). After 3 washes with PBS, sections were incubated in Cy3-conjugated goat anti-rabbit antibody (1:500; Jackson ImmunoResearch Inc., West Grove, Pennsylvania). Pictures of mounted sections were taken using MetaMorph Imaging System (Molecular Devices, Downingtown, Pennsylvania) and the intensity of the fluorescent stain within the striatum was measured. 10 WT, 12 YAC128, 10 YAC18 and 8 YAC18/128 were used for this analysis.

Immunohistochemistry for markers of neuronal health was performed as described above using the following antibodies: synaptophysin (1:100; BD Transduction Laboratories, Mississauga, Ontario), calbindin (1:2000; provided by Ken Bainbridge, University of British Columbia, Canada), EM48 (1:500; provided by Xiao-Jiang Li, Emory University, U.S.A) and 8-hydroxy-2-deoxyguanosine (1:500; Japan Institute for the Control of Aging, Fukuroi City, Japan).

### Statistical analysis

Overall effects of genotype were determined by one way ANOVA. Repeated measures ANOVA analysis was used for analysis of differences in rotarod performance. The significance of differences between YAC128 and YAC18/128 mice was determined by either the Tukey post-hoc test following ANOVA or a student's t-test.

## Authors' contributions

JVR conceived the study, designed the experiment, completed the neuropathological assessment of the mice except for the striatal DARPP-32 levels, carried out the Western blot for human htt, prepared the figures for the manuscript and wrote the manuscript. JP carried out the behavioural analysis including rotarod and open field testing. ZM completed the western blot for total htt. MRH and BRL contributed to the conception and design of the experiment and were also involved in editing the manuscript. All of the authors have read and approved the final manuscript.

## Supplementary Material

Additional File 1 Supplemental Table 1. This table summarizes the effect of increasing or decreasing wild-type huntingtin levels on phenotypic severity in the YAC128 mouse model of Huntington disease Click here for file

## Figures and Tables

**Table 1:** Effect of modulating wild-type huntingtin levels on survival in YAC128 mice.

Sex	Genotype	Deaths	Total Mice	Percent Surviving
Males	WT	4	46	91%
	**YAC18/128**	**3**	**11**	**73%**
	YAC128	12	48	75%
	YAC128-/-	11	15	27%

Females	WT	5	42	88%
	**YAC18/128**	**0**	**4**	**100%**
	YAC128	5	66	92%
	YAC128-/-	3	19	84%

YAC128-/- mice express mutant htt and no wild-type htt. YAC128 mice express mutant htt and 2 copies of wild-type htt. YAC18/128 express mutant htt and increased levels of wild-type htt.

**Table 2:** Effect of over-expression of wild-type huntingtin on HD-like phenotypes in the YAC128 mouse model of HD.

**Phenotype**	**Percent Difference YAC128 Compared to WT**	**Percent Difference YAC18/128 Compared to WT**	**Percent Rescue with WT htt Expression**	**Significance**
Rotarod	-36%	-41%	None	p = 0.5
Activity – 2 Months	+6%	+2%	74%	P = 0.5
Activity – 12 Months	-10%	-6%	47%	P = 0.5
Striatal Volume	-9%	-7%	21%	p = 0.3
Striatal Neuronal Counts	-6%	-3%	38%	p = 0.4
Striatal DARPP-32 Expression	-18%	-14%	22%	p = 0.3
Striatal Neuronal Size	-12%	-2%	87%	p < 0.001
